# Levels and values of circulating endothelial progenitor cells, soluble angiogenic factors, and mononuclear cell apoptosis in liver cirrhosis patients

**DOI:** 10.1186/1423-0127-19-66

**Published:** 2012-07-18

**Authors:** Chih-Hung Chen, Li-Teh Chang, Wei-Chih Tung, Yung-Lung Chen, Chia-Lo Chang, Steve Leu, Cheuk-Kwan Sun, Tzu-Hsien Tsai, I-Ting Tsai, Hsueh-Wen Chang, Hon-Kan Yip

**Affiliations:** 1 Divisions of General Medicine, Kaohsiung Chang Gung Memorial Hospital and Chang Gung University College of Medicine, Kaohsiung, Taiwan; 2 Basic Science, Nursing Department, Meiho University, Pingtung, Taiwan; 3 Division of Hepato-gastroenterology, Kaohsiung Chang Gung Memorial Hospital and Chang Gung University College of Medicine, Kaohsiung, Taiwan; 4 Division of Cardiology, Department of Internal Medicine, Kaohsiung Chang Gung Memorial Hospital and Chang Gung University College of Medicine, 123, Ta Pei Road, Niao Sung Hsiang, Kaohsiung Hsien, 83301, Taiwan; 5 Division of Colorectal Surgery, Department of Surgery, Kaohsiung Chang Gung Memorial Hospital and Chang Gung University College of Medicine, Kaohsiung, Taiwan; 6 Center for Translational Research in Biomedical Sciences, Kaohsiung Chang Gung Memorial Hospital and Chang Gung University College of Medicine, Kaohsiung, Taiwan; 7 Department of Emergency Medicine, E-Da Hospital, I-Shou University, Kaohsiung, Taiwan; 8 Department of Biological Sciences, National Sun Yat-Sen University, Kaohsiung, Taiwan

**Keywords:** Liver cirrhosis, Circulating endothelial progenitor cells, Angiogenesis factors, Cellular apoptosis

## Abstract

**Background:**

The roles of circulating endothelial progenitor cell (EPC) and mononuclear cell apoptosis (MCA) in liver cirrhosis (LC) patients are unknown. Moreover, vascular endothelial growth factor (VEGF) and stromal cell-derived factor (SDF)-1α are powerful endogenous substances enhancing EPC migration into circulation. We assessed the level and function of EPCs [CD31/CD34 (E_1_), KDR/CD34 (E_2_), CXCR4/CD34 (E_3_)], levels of MCA, VEGF and SDF-1α in circulation of LC patients.

**Methods:**

Blood sample was prospectively collected once for assessing EPC level and function, MCA, and plasma levels of VEGF and SDF-1α using flow cytometry and enzyme-linked immunosorbent assay (ELISA), respectively, in 78 LC patients and 25 age- and gender-matched healthy controls.

**Results:**

Number of EPCs (E_1_, E_2_, E_3_) was lower (all p < 0.0001), whereas SDF-1α level and MCA were higher (p < 0.001) in study patients compared with healthy controls. Number of EPCs (E_2_, E_3_) was higher but MCA was lower (all p < 0.05) in Child's class A compared with Child's class B and C patients, although no difference in VEGF and SDF-1α levels were noted among these patients. Chronic hepatitis B and esophageal varices bleeding were independently, whereas chronic hepatitis C, elevated aspartate aminotransferase (AST), and decompensated LC were inversely and independently correlated with circulating EPC level (all p < 0.03). Additionally, angiogenesis and transwell migratory ability of EPCs were reduced in LC patients than in controls (all p < 0.001).

**Conclusion:**

The results of this study demonstrated that level, angiogenic capacity, and function of circulating EPCs were significantly reduced, whereas plasma levels of SDF-1α and circulating MCA were substantially enhanced in cirrhotic patients.

## Background

Since multitudinous studies have shown that circulating endothelial progenitor cells (EPCs) are essential for neoangiogenesis and the endothelial repair process, they have been employed for the treatment of both experimental and clinical organ ischemia in various settings over the past few years [1-6]. Growing data have demonstrated that EPC migration into circulation and homing to ischemic tissues/organs are strongly associated with an increase in circulating levels of chemokines [7], cytokines [8], recombinant human erythropoietin (EPO) [9], and some specific medications such as statins [10] or angiotensin converting enzyme inhibitors (ACEIs) [11], as well as extracorporeal shock wave therapy [12]. Additionally, angiogenesis in malignant tumors and metastasis have also been reported to be associated with an increased number of circulating EPCs [13,14]. Besides, vascular endothelial growth factor (VEGF) and stromal cell-derived factor-1 alpha (SDF-1α), two powerful soluble angiogenesis factors, have been shown to play a crucial role in enhancing EPC migration from bone marrow into circulation [15]. Conversely, decreased circulating number of EPCs has been found to correlate to the prevalence of risk factors for coronary artery disease [16,17], recurrent ischemic stroke [18], and advanced heart failure [19].

Liver cirrhosis (LC) of divergent etiologies is at high risk of developing into hepatocellular carcinoma (HCC) [20,21]. Moreover, abundant studies have shown that not only serum levels of soluble angiogenic factors are notably increased in patients with LC and those with HCC, but they are also useful biomarkers for predicting the prognostic outcome in these patient populations [22-24]. Surprisingly, the role of circulating EPCs in the clinical setting of LC has seldom been investigated [25]. Besides, the link between circulating number of EPC and plasma level of VEGF and SDF-1α is currently unclear in LC patients. Furthermore, although progression of chronic liver disease to LC is known to involve distinctive apoptosis and death of hepatocytes, there is a lack of in-depth scrutiny regarding the role of circulating mononuclear cell apoptosis (MCA) in modulating the severity of chronic liver disease. Of particular importance is that the function of EPC in LC patients has not been reported. In this study, the above issues were addressed by applying flow cytometric and ELISA analysis to determine the number and function of EPCs, the distinctive role of circulating MCA, and the relationship between EPC biomarkers and plasma levels of VEGF and SDF-1α in LC patients.

## Methods

### Patient enrollment and blood sampling

The estimated sample size of 86 patients was based on the effective size with an α value of 0.05, a power of 80 %, a 0.7 % difference in circulating level of EPCs between study patients and healthy control subjects, and standard deviations of 0.7 % in normal control subjects and 1.0 % in study patients. A 12.0 % rate of protocol violations was assumed. The calculation of sample size for specific objective was based on our recent report [18]. The expected 0.7 % was based on our previous study of EPC surface marker of CD31/CD34 on 138 patients [18].

To circumvent other potential influences on measurement of circulating level of EPCs, patients with one or more of the followings were excluded: recent surgery or trauma during the preceding 2 months, malignancy, history of febrile disorders, acute or chronic inflammatory disease other than LC during the study period, history of autoimmune diseases with or without immunosuppressive therapy, and prior myocardial infarction with an onset of < 3 months.

Between September 2009 and December 2010, 88 patients who presented to the Department of Gastroenterology at Kaohsiung Chang Memorial Hospital with the diagnosis of LC were recruited. Patients were enrolled either from out patient department (n = 7, all of them were Child’s class A patients) (followed-up for LC) or during hospitalization (n = 71) for severe jaundice, ascites with hypoalbuminemia for plasma transfusion, acute aspartate aminotransferase (AST) and alanine aminotransferase (ALT) elevation, anti-hepatitis B (entecavir) treatment, anti-hepatitis C (interferon) treatment, esophageal varices ligation or esophageal varices bleeding (n = 10). After excluding 10 patients due to infection or refusing to participate in the study, blood sampling was performed in totally 78 consecutive patients. All blood sampling was collected prior to blood transfusion.

Twenty-five age- and gender-matched healthy controls were also studied. Informed consent was obtained from all study subjects. The study protocol was approved by the Institutional Review Committee on Human Research at Kaohsiung Chang Gang Memorial Hospital.

### Subgroup analysis of the patients

According to the degree of decompensation in liver function, the study patients were categorized into Child's class A, B, and C to investigate whether the circulating levels of EPCs, MCA, and plasma levels of SDF-1α and VEGF were different among these patients.

### Ultrasonographic examination and definitions

Ultrasonographic scans were performed by experienced hepatologists at our institute using an ultrasound system with a 3.5 MHz convex probe (SSA-340, SSA-370, Aplioxu-700 Toshiba, Tokyo, Japan; SSD 2000, Aloka, Tokyo, Japan; HDI 5000, ATL Ultrasound, Bothell, USA; Preirus Hi-vision Hitachi, Tokyo, Japan).

Chronic hepatitis C was defined as detectable serum antibody to hepatitis C virus (anti-HCV), and chronic hepatitis B was defined as detectable serum hepatitis B viral surface antigen (HBsAg). The definitions and diagnoses of HCC, LC, and the degree of decompensation were based on previous reports [26-30].

### Blood sampling and assessment of circulating EPC level by flow cytometry

Blood samples were obtained once in ward or in the outpatient department for the study group and also once for the healthy control subjects. Twenty milliliters of blood was drawn from the antecubital vein (10 mL for EPC analysis and another 10 mL for MCA assessment). Mononuclear cells (MNCs) were then isolated by density-gradient centrifugation of Ficoll 400 (Ficoll-Plaque^TM^ plus, Amersham Biosciences, Sweden) as previously described [31]. The isolated MNCs were washed twice with phosphate buffer solution (PBS) and centrifuged before incubation with 1 mL blocking buffer (BSA) for 30 minutes at 4 ^0^ C. Cell viability of > 95.0 % was noted in each group.

It has been reported that EPCs are characterized by surface expression of CD34, CD31, CXCR4 and kinase insert domain-conjugating receptor (KDR) antigens [9,12,18,19,31,32]. In this study, EPCs were defined as cells with co-expression of CD34, endothelial cell (EC) lineage-markers [KDR, vascular endothelial (VE)-cadherin, CD31, and CXCR4 according to previous reports [9,12,18,19,31,32] and our recent study [18].

EPCs in peripheral blood were identified by flow cytometry using the technique of double staining as depicted in our recent report [18]. To determine the EPC surface markers of CD31/CD34 (E_1_), KDR/CD34 (E_2_) and CXCR4/CD34 (E_3_), MNCs (4 x 10^5^) were incubated for 30 minutes at 4 ^0^ C in a dark room with monoclonal antibodies against KDR (Sigma), fluorescein isothiocyanate (FITC)-conjugated CD34, phycoerythrin (PE)-conjugated CD31, and CXCR4 (Becton Dickinson). Control ligand (IgG-PE conjugate) was used to detect possible nonspecific association and define a threshold for glycoprotein binding. For analysis of KDR, MNCs were further incubated with goat anti-mouse antibody conjugated with PE. After staining, the MNCs were fixed in 1 % paraformaldehyde. Quantitative two-color flow cytometric analysis was performed using a fluorescence-activated cell sorter (FACSCalibur^TM^ system; Beckman). Each analysis included 30,000 cells (i.e., mononuclear cells) per sample. The assays for EPCs (E_1-3_) in each sample were performed in duplicate and the mean levels were reported. The results were expressed as percentage (%) of total mononuclear cells per sample.

Intra-assay variability based on repeated measurement of the same blood sample was low with a mean coefficient of variance of 4.6 % in LC patients and 4.1 % in healthy control subjects.

### Mononuclear cell culture, differentiation of endothelial cell phenotype, angiogenesis, and measurement of total tubular length

To evaluate the degree of angiogenesis, another 10 mL of blood was drawn randomly from 6 LC patients (2 in Child’s Class A, B and C, respectively) and 6 normal subjects with age match, respectively. The protocol and procedure of cell culture and the determination of angiogenesis were based on our recent report [33]. Briefly, the MNCs were isolated by density-gradient centrifugation of Ficoll 400, followed by cultivation in differential endothelial cell culture medium (endothelial cell basal medium-2, Cambrex) with 10 % fetal bovine serum (FBS), 50 U/mL penicillin, 50 μg/mL streptomycin and 2 mmol/L L-glutamine (Invitrogen) with VEGF and basic fibroblast growth factor (10 ng/ml) plated on gelatin-coated tissue culture flasks and incubated at 37 ^0^ C with 5 % CO_2_ for 21 days. Culture medium was changed every 48 hours. By day 21, cells with spindle-shaped and cobblestone-like phenotype typical of endothelial cells were found attached on the plate.

The cells with endothelial cell phenotype were then plated in 96-well plates at 1.0 × 10^4^ cells/well in 150 μL serum-free M199 culture medium mixed with 50 μL cold Matrigel (Chemicon international) for 24 hours using passage 2 EPCs incubated at 37 °C in 5 % CO_2_. Three random microscopic images (200 x) were taken from each well for counting cluster, tube, and network formations with the mean values obtained. Both cumulative and mean tube lengths were calculated by Image-Pro Plus software (Media Cybernetics).

### Transwell migratory assay

To investigate the transwell migratory ability of EPCs (n = 6 in each experiment), transwell membranes (5 μm; Costar, Germany) were coated on both sides with fibronectin (2.5 μg/mL; Roche, Mannheim, Germany) overnight at 4 °C. The experimental protocol was also based on that of our recent report [32]. EPCs (1 x 10^5^ cells/well) were resuspended in M199 medium (Gibco, Carlsbad, CA, USA) containing 0.5 FBS (Gibco, Carlsbad, CA, USA) and incubated in the upper chamber at 37 °C in 5 % CO_2_ and allowed to migrate for 18 hours toward the lower chamber which was filled with M199 containing 20 % FBS. Cells remaining on the upper surface of the transwell membranes were mechanically removed and cells that had migrated to the lower surface were fixed with 4 % formaldehyde. For cell quantification, nuclei of the migrated cells were stained with DAPI. Cells migrating into the lower chamber were counted in 5 random microscopic fields using a fluorescence microscope (Olympus, Tokyo, Japan) with the software Image-Pro Plus (Media Cybernetics, Bethesda, MD, USA).

### Determination of annexin V level by flow cytometry

Flow cytometric analysis using double staining of annexin V and propidium iodide (PI) is a simple and popular method for the identification of apoptotic cells [i.e. early phase (annexin V+/PI-) and late phase (annexin V+/PI+) of apoptosis].

Ten milliliters of peripheral blood MNCs were isolated by density gradient centrifugation of Ficoll 400 (Ficoll-Plaque^TM^ plus, Amersham Biosciences, Sweden). Cell viability of > 95.0 % was noted in each group. The protocol and procedure were based on our recent report [33].

The annexin V kit (Pharmingen, Becton Dickinson, San José, CA, USA) was used for apoptosis analysis according to the manufacturer’s instructions. The supernatant was decanted and the pellet was resuspended in 200 μL of 1x annexin V binding buffer. Cells were incubated with 2 μL of annexin V-FITC and 5 μL of propidium iodide (PI) for 15 minutes at room temperature in the dark. Finally, 500 μL of 1x annexin V Binding Buffer was added, followed by immediate flow cytometric analysis. Identical to the analysis of EPCs, each analysis included 30,000 cells (i.e., mononuclear cells) per sample. The assays for MCA in each sample were performed in duplicate and the mean levels were reported. The results were expressed as percentage (%) of total mononuclear cells per sample.

Intra-assay variability based on repeated measurements of the same blood sample was low with a mean coefficient of variance less than 4.2 % in both study patients and normal subjects.

### Measurement of plasma levels of VEGF and SDF-1α using ELISA

Plasma concentrations of VEGF and SDF-1α were assessed using VEGF and SDF-1α antibody ELISA kit (R&D), respectively according to the manufacturer's protocol.

### Statistical analysis

Data were expressed as means ± SD. Continuous data between two groups were analyzed using Wilcoxon rank sum test, and categorical data were analyzed by chi-square or Fischer exact test. For determining statistical significance among the three groups, continuous data were analyzed using Kruskal-Wallis test, followed by Wilcoxon rank sum test with Bonferroni’s correction. Statistical analysis was performed using SAS statistical software for Windows version 8.2 (SAS institute, Cary, NC). A p value of < 0.05 was considered statistically significant.

## Results

Table 1 shows the baseline characteristics and laboratory findings of study patients and healthy control subjects. The age, gender, white blood cell (WBC) count, and serum creatinine level did not differ between the two groups. However, the circulating level of EPCs [CD31/CD34(E_1_), KDR/CD34(E_2_), CXCR4/CD34(E_3_)], hemoglobin level, platelet count, and serum albumin concentration were remarkably lower in study patients than those in control subjects. By contrast, the serum levels of AST, ALT, and plasma level of SDF-1α were substantially increased in study patients compared with those in control subjects, whereas plasma VEGF level was similar between the two groups. Additionally, flow cytometry revealed a notable enhancement of early and late MCA in LC patients than in healthy control subjects in the present study (Table 1).

**Table 1:** The baseline Characteristics of Study Patients and Normal Controls

**Variables**	**Study patients (n = 78)**	**Normal control (n = 25)**	p value*
Age (yrs)	54.8 ± 13.7	54.1 ± 10.5	0.927
Male gender	71.8 % (56)	68.0 % (17)	0.716
EPC surface markers†			
CD31/CD34 (%)‡	1.01 ± 0.57	1.37 ± 0.49	0.005
KDR/CD34 (%)‡	1.08 ± 0.63	1.37 ± 0.65	0.035
CXCR4/CD34 (%)‡	1.13 ± 0.66	1.54 ± 0.64	0.001
WBC count (x 10^3^/dl)	8.5 ± 11.9	6.0 ± 1.5	0.575
Hemoglobin (g/dl)	10.9 ± 2.4	14.9 ± 1.3	<0.0001
Platelet count (x 10^4^/dl)	10.8 ± 6.3	22.4 ± 5.5	<0.0001
Creatinine (mg/dl)	1.00 ± 0.60	0.91 ± 0.20	0.806
Albumin (g/dl)	3.0 ± 0.6	4.5 ± 0.3	<0.0001
AST (U/L)	97.8 ± 107.1	24.6 ± 6.4	<0.0001
ALT (U/L)	68.1 ± 123.0	29.3 ± 17.4	0.005
VEGF (pg/ml)	55.4 ± 58.0	49.9 ± 27.8	0.286
SDF-1α (pg/ml)	2256.3 ± 705.8	1502.4 ± 370.7	<0.0001
Early apoptosis (%)	18.01 ± 11.66	6.16 ± 2.83	<0.0001
Late apoptosis (%)	3.28 ± 3.86	1.19 ± 0.99	0.0008

Data expressed as mean ± SD or % (No).

ALT = alanine aminotransferase; AST = aspartate aminotransferase; EPC = endothelial progenitor cell; SDF = stromal cell-derived factor; VEGF = vascular endothelial cell growth factor; WBC = white blood cell.

* by Wilcoxon rank sum test.

† indicates EPC surface markers with double stains.

‡ The expected 0.7 % was based on our previous study [18] on EPC surface markers (CD31/CD34). The expected difference (i.e., 0.7 %) has not been reached for CD31/CD34 (or KDR/CD34, CXCR4/CD34) in Table 1.

The incidences of male gender, diabetes mellitus, hypertension, and current smoking did not differ among Child's class A, B and C patients (Table 2). The incidences of chronic hepatitis B, chronic hepatitis C, history of esophageal varices (EV) bleeding, ascites on sonographic examination, and portal venous hypertension were similar among the three groups of patients. However, patients in the Child's class C were younger than the other two groups. Additionally, the incidences of decompensated LC and portal venous hypertension were remarkably higher in patients in Child's class C than those in Child's class A and B, and notably higher in patients in Child's class B than those in Child's class A. Furthermore, the incidences of current smoking and splenomegaly were significantly higher in Child's class B and C patients than those in Child's class A, but they showed no difference between those in Child's class B and C.

**Table 2:** Clinical Characteristics and Abdominal Ultrasonographic Findings of 78 Study Patients

**Variables**	**Child’s class A (n = 9)**	**Child’s class B (n = 26)**	**Child’s class C (n = 43)**	p value*
Age (yrs)	59.9 ± 11.3 ^a^	59.6 ± 14.4 ^a^	50.8 ± 12.7 ^b^	0.030
Male gender	55.6 % (5)	65.% (17)	79.1 % (34)	0.234
Diabetes mellitus	33.3 % (3)	46.2 % (12)	20.9 % (9)	0.089
Hypertension	22.2 % (2)	11.5 % (3)	2.3 % (1)	0.0503
Current smoking	11.1 % (1)^a^	73.1 % (19)^b^	48.8 % (21)^b^	0.006
Chronic hepatitis B	55.6 % (5)	38.5 % (10)	27.9 % (12)	0.251
Chronic hepatitis C	11.1 % (1)	30.8 % (8)	10 (23.3 %)	0.513
Previous EV bleeding	22.2 % (2)	30.8 % (8)	46.5 % (20)	0.295
Hepatocellular carcinoma	0 % (0)	23.1 % (6)	14.0 % (6)	0.286
Liver cirrhosis	100.0 % (9)	100.0 % (26)	100.0 % (43)	1.000
Ascitis	22.2 % (2)	50.0 % (13)	58.1 % (25)	0.136
Decompensated liver cirrhosis	11.1 % (1)^a^	38.5 % (10)^b^	67.4 % (29)^c^	0.002
Splenomegaly	22.2 % (2)^a^	76.9 % (20)^b^	67.4 % (29)^b^	0.015
Portal vein hypertension	11.1 % (1)^a^	30.8 % (8)^b^	53.5 % (23)^c^	0.028

Data expressed as mean ± SD or % (No).

EV, esophageal varices.

*Continuous data were analyzed using Kruskal-Wallis test; categorical data were analyzed using Chi-square or Fisher’s exact test appropriately. Letters (^a, b, C^) indicate significant difference (at 0.05 level) (by Wilcoxon rank sum test with Bonferroni’s correction).

Flow cytometric analysis (Table 3) demonstrated that the circulating number of E_2_ and E_3_ was significantly increased in Child's class A patients than those in Child's class B and C, but the number of E_1_, E_2_ and E_3_ did not differ between those in Child's class B and C.

**Table 3:** Flow Cytometric Analysis and Laboratory Findings of 78 Study Patients

**Variables**	**Child’s class A (n = 9)**	**Child’s class B (n = 26)**	**Child’s class C (n = 43)**	p value*
EPC surface makers (double stains)				
CD31/CD34 (%)	1.23 ± 0.44	0.90 ± 0.52	1.03 ± 0.61	0.279
KDR/CD34 (%)	1.60 ± 0.51^a^	0.97 ± 0.63^b^	1.03 ± 0.61^b^	0.013
CXCR4/CD34 (%)	1.49 ± 0.56^a^	1.20 ± 0.63^b^	1.00 ± 0.61^b^	0.043
Early apoptosis (%)	13.5 ± 8.9	16.8 ± 10.8	19.7 ± 12.5	0.281
Late apoptosis (%)	1.66 ± 1.62^a^	3.12 ± 4.50^b^	3.67 ± 3.7^b^	0.046
Hemoglobin (g/dl)	12.2 ± 2.2	10.1 ± 2.5	11.1 ± 2.2	0.110
WBC count (x 10^3^/dl)	5.0 ± 2.1	8.7 ± 12.5	8.8 ± 12.3	0.700
Platelet count (x 10^4^/dl)	13.6 ± 11.9	11.0 ± 5.5	10.3 ± 5.7	0.930
Creatinine (mg/dl)	0.84 ± 0.23	0.93 ± 0.34	1.08 ± 0.74	0.805
Albumin (g/dl)	2.54 ± 0.33^a^	2.89 ± 0.54^b^	2.87 ± 0.58^b^	0.005
AST (U/L)	50.0 ± 23.6	114.0 ± 152.1	98.0 ± 80.4	0.097
ALT (U/ml)	45.0 ± 32.5	83.6 ± 195.2	63.5 ± 67.7	0.361
Prothrombin time (sec)	11.0 ± 0.7^a^	13.6 ± 3.9^b^	13.2 ± 2.2^b^	0.006
α-fetoprotein (ng/ml)	8.3 ± 3.2	75.8 ± 223.5	1187.5 ± 6495.3	0.430
Total bilirubin (mg/dl)	0.8 ± 0.2^a^	3.4 ± 3.2^b^	4.8 ± 6.4^b^	0.0002
VEGF (pg/ml)	25.0 ± 11.4^a^	54.1 ± 22.4^b^	62.6 ± 59.8^b^	0.039
SDF-1α (pg/ml)	2030.1 ± 446.4	2246.1 ± 808.0	2309.8 ± 687.6	0.350

Data expressed as mean ± SD or % (No).

ALT = Alanine aminotransferase; AST = aspartate aminotransferase; SDF = stromal cell-derived factor; VEGF = vascular endothelial cell growth factor; WBC = white blood cell.

* Continuous data were analyzed using Kruskal-Wallis test. Letters (^a, b^) indicate significant difference (at 0.05 level) (by Wilcoxon rank sum test with Bonferroni’s correction).

To elucidate the impact of decompensation of liver function on MCA in circulation, flow cytometric analysis using the technique of double staining for annexin V and PI was performed for each patient. The results showed that early MCA was similar among the three groups of the patients, whereas late MCA was substantially lower in those in Child's class A than those in Child's class B and C. No significant difference, however, was noted between patients in Child's class B and C.

Laboratory findings demonstrated no significant differences in hemoglobin, WBC count, platelet count, serum levels of creatinine, AST, ALT, and α-fetoprotein among the three groups of patients. On the other hand, prothrombin time and serum level of total bilirubin were significantly lower in Child's class A than in Child's class B and C patients. These two parameters, however, did not differ between Childs' class B and C patients. Paradoxically, serum albumin level was notably lower in Child's class A than in Child's class B and C patients.

The plasma level of SDF-1α was similar among the three groups of patients. In addition, although no significant difference in plasma level of VEGF was noted between Child's class B and C patients, it was significantly lower in Child's class A than in Child's class B and C patients.

**Figure 1:**
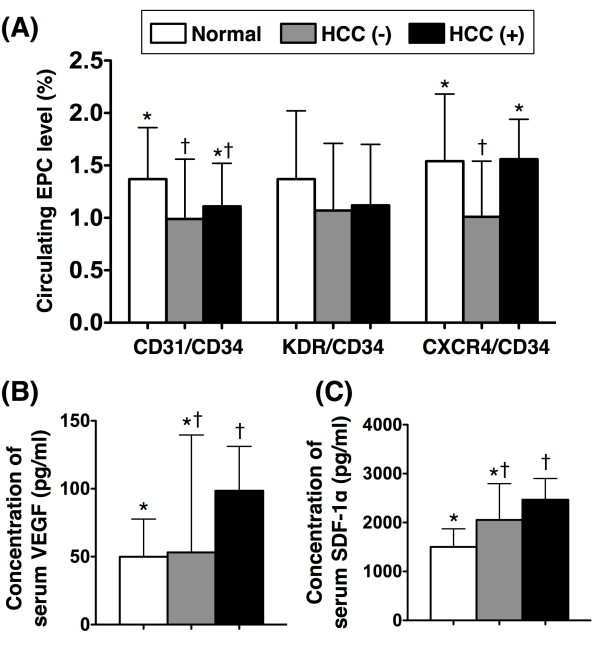
**Flow Cytometric and ELISA Analysis among Patients With and Without HCC and in Normal Control Subjects. A**) Circulating number of endothelial progenitor cells (EPCs). For CD31/CD34+ cells, * vs. †, p < 0.04. For CXCR4+/CD34+ cells, * vs. †, p < 0.03*.* HCC (−) = without hepatocellular carcinoma (n = 66); HCC (+) = with hepatocellular carcinoma (n = 12). **B**) Plasma level of vascular endothelial growth factor (VEGF). * vs. †, p < 0.035. **C**) Plasma level of stromal cell-derived factor (SDF)-1α. * vs. †, p < 0.04. Symbols (*, †) indicate significant difference (at 0.05 level) (by Wilcoxon rank sum test with Bonferroni’s correction).

To compare the number of circulating EPCs and plasma levels of VEGF and SDF-1α among LC patients with (n = 66) or without HCC (n =12) and the healthy control (n = 25) subjects, subgroup analysis was performed (Figure 1A). The number of circulating E_2_ did not differ among LC patients and healthy controls. Additionally, the numbers of circulating E_1_ were similar between LC patients with or without HCC. Furthermore, the number of circulating E_1_ did not differ between LC with HCC and healthy controls, but it was significantly lower in LC patients without HCC than in healthy controls. Moreover, the circulating number of E_3_ did not differ between LC patients with HCC and healthy control subjects. Interestingly, this biomarker (E_3_) in LC patients without HCC was not only notably lower than in healthy subjects but it was also significantly lower than in LC patients with HCC (Figure 1A). The plasma level of VEGF showed no difference either between LC patients with and without HCC, or between LC patients without HCC and healthy subjects. This biomarker, however, was notably elevated in patients with HCC compared with that in healthy subjects (Figure 1B). Additionally, the plasma level SDF-1α did not differ between LC patients with or without HCC, or between LC patients without HCC and healthy subjects, but it showed significantly higher in LC patients with HCC than in healthy subjects (Figure 1C). These findings imply that LC patients with HCC might have higher ability than those with LC alone to secrete SDF-1α and VEGF that, in turn, enhanced the mobilization of EPCs from bone marrow to circulation.

To identify the independent predictors of increased circulating level of EPCs (Table 4), a number of enrollment variables were utilized (Tables 2 and 3). Multiple linear regression analysis demonstrated that chronic hepatitis B and EV bleeding were significantly and independently associated with increased circulating level of EPCs. By contrast, hepatitis C, decompensated LC, and serum AST level were inversely and independently predictive of decreased circulating level of EPCs.

**Table 4:** Multiple Stepwise Linear Regression Analysis of Independent Predictors of Increased Circulating Level of EPCs*

**Variables**	**CD31/CD34**		**KDR/CD34**		**CXCR4/CD34**	
	**Coefficient β**	**p value**	**Coefficient β**	**p value**	**Coefficient β**	**p value**
Age	−0.009	0.059				
Hepatitis B	0.340	0.011	0.380	0.008		
Hepatitis C			−0.332	0.035		
EV bleeding			0.291	0.028		
Liver cirrhosis†	0.352	0.087			−0.708	0.003
AST			−0.001	0.006		

AST = aspartate aminotransferase; EPC = endothelial progenitor cells; EV = esophageal varicis.

* Variables of Tables 2 and 3 were enrolled for analysis.

† indicated decompensated liver cirrhosis.

**Figure 2:**
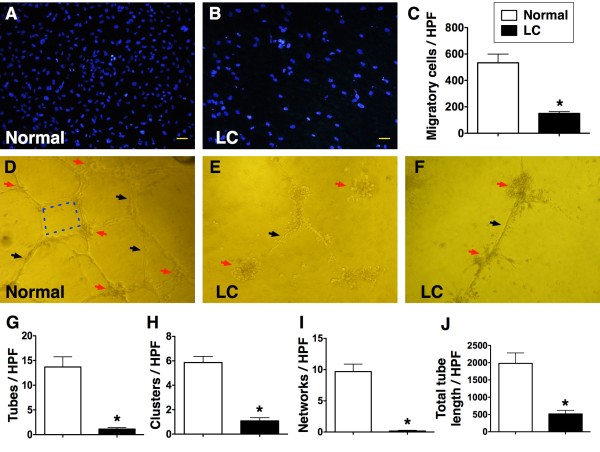
**In vitro function assessment of endothelial progenitor cells with transwell and matrigel tube formation assay. A** to **C**) The transwell migratory activity of endothelial progenitor cells (EPCs) (100 x) was notably reduced (C) in liver cirrhosis (LC) group than in healthy control group (* vs. normal control, p < 0.0001) (n = 6). **D to F**). Illustration of angiogenesis using passages 2 of culturing cells (all pictures were observed under microscope of 400 x). The cluster formation (red arrows), tubular formation (black arrows) and network formation (blue dot lines) were notably higher in normal control than in LC patients (**G to J**). (* vs. normal control, all p values < 0.001) (n = 6) High-power field (HPF).

To compare the angiogenesis ability and EPC function between study patients and healthy control subjects, MNC culture for EPC was performed (Figure 2). Interestingly, the results showed that the cluster formation (p < 0.001), tubular formation (p < 0.0001), and network formation (p < 0.0001) were remarkably lower in LC patients than in healthy control subjects. In addition, the accumulative tubular length (P < 0.0001) was notably reduced in LC patients than in healthy control subjects. Furthermore, the number of transwell migratory EPCs was substantially lower in LC patients as compared with those in healthy control subjects (p < 0.0001).

## Discussion

Several striking implications were noted in this study that investigated the number and function of circulating of EPCs, plasma levels of VEGF, SFD-1α, and MCA in circulation. First, the circulating levels of SDF-1α as well as the degree of both early and late MCA were remarkably higher in LC patients compared with those in healthy control subjects. Second, the circulating number of EPCs was notably lower in LC patients than that in normal control subjects. Third, the circulating number of EPCs was markedly decreased, whereas the degree of late MCA in circulation was significantly increased in Child’s class B and C patients compared with those in Child’s class A.

Surprisingly, while the association between increased serum levels of soluble angiogenic factors and chronic liver disease and HCC has been extensively investigated, circulating EPC level in LC patients has seldom been reported [25]. One important finding in the current study is that the number of circulating EPCs was notably reduced in patients with LC compared to that in age- and gender-matched healthy control subjects. Of particularly interest is that, when compared with Child’s class A patients, this biomarker was further decreased in those in Child’s class B and C. Our results, therefore, show that the degree of decompensation in liver function was independently correlated to the reduction in circulating number of EPCs (Table 4). Interestingly, Yu et al.. has recently demonstrated dynamic changes in the levels of circulating EPCs in cirrhotic liver using a rat model of HCC [34]. The results of their experiment revealed a gradual increase in the number of circulating EPCs up to 4 weeks after the onset of liver disease, followed by a gradual drop to less than the baseline level at more advanced stage of LC [34]. Accordingly, our findings corroborate those from Yu et al.[34]. Of importance was that our findings may, therefore, encourage the use of circulating EPC number as an accessory tool in our clinical practice for evaluating the severity of decompensation in liver function.

Although elevations in the serum levels of soluble angiogenic factors are well-documented in the setting of advanced liver disease as in LC and HCC, the reason for the reduction in circulating number of EPCs in LC patients compared with that in healthy control subjects remains unclear. The results of the present study provided several possible explanations for the phenomenon. First, since the progression from fibrosis to cirrhosis, the end-point of chronic liver diseases, is characterized by a chronic and persistent inflammatory reaction [35.36], we suggest that such chronic inflammation, in addition to stimulating the local release of angiogenic factors by hepatic stellate cells and sinusoidal endothelial cells [35,36], may at the same time suppress the mobilization of EPCs from bone marrow into the circulation. Our hypothesis is supported by the findings of previous studies [16,17] showing a significant decrease in the circulating number of EPCs in patients with hypertension, hypercholesterolemia, current smoking, and diabetes mellitus that are well-known risk factors for coronary artery disease and indexes of chronic inflammatory disease. Second, substantial reduction in circulating EPCs has also reported in patients with decompensated heart failure [19] that is believe to be attributable to an exhaustion of the sources of EPCs. We propose that decompensated heart failure and decompensated LC are two sides of the same coin. Consistently, 50 % of our patients have been identified to have decompensated LC. This proposed mechanism is supported by the findings from both experimental [34] and clinical observational [19] studies.

Surprisingly, contrary to the findings of the present study, a recent investigation [25] demonstrated a higher circulating level of EPCs (i.e. triple staining of the surface EPC marker: CD34/KDR/AC133) in patients with HCC than in those with LC. Moreover, that study also showed a higher number of circulating EPCs in LC patients than that in normal controls. Although the discrepancy in results between ours and those from that study [25] seems paradoxical, there may be three possible explanations. First, the treatment status of the patients was different in the two studies. While the previous report enrolled patients waiting for HCC treatment, the current study recruited patients with HCC after treatment, including percutaneous ethanol injection, radiation therapy, radiofrequency ablation, liver lobectomy, or chemotherapy. Second, the degree of decompensation in liver function and the staging for advanced LC may not be identical between the two studies. Finally, differences in sample size, exclusion criteria, methodology for identification of EPCs (CD34/KDR/AC133 in the recent study [25] vs. CD31/CD34, KDR/CD34 and CXCR4/CD34 in the present study) may also contribute to the discrepancy in results between the two studies. Another interesting finding is that, as compared with LC patients without HCC, those with HCC had significantly higher circulating number of E_3_. Besides, LC patients with HCC also had notably higher plasma level of VEGF compared to that in healthy control subjects. In terms of subgroup analysis, therefore, our results were comparable to those of the recent report [25].

The role of SDF-1α has been established as the ligand of CXCR4+ cells. It has also been reported to play an essential role in mobilizing EPCs from bone marrow to circulation to reach ischemic tissue/organ for angiogenesis and organ repair/regeneration [15]. According to a recent report from Hong et al. [35], activation of CXCR4 receptor by SDF-1*α* is profibrogenic in the liver through activation of hepatic stellate cells. In addition to the elevated number of circulating cells with E_3_ in LC patients with HCC than in LC patients without HCC, another interesting finding in the present study is the substantial increase in plasma level of SDF-1α in LC patients with HCC compared to that in those LC patients without HCC and healthy subjects. Therefore, the results from this clinical observational investigation further strengthen those from Hong’s in vivo and in vitro studies [35].

The most important finding in the current study is that, compared with that in the healthy subjects, the capacity for angiogenesis (i.e. cluster, network and tubular formations) was markedly impaired in LC patients. Besides, the transwell migratory ability of EPCs was significantly reduced in LC patients compared to that of the healthy controls. These novel findings implicate that not only was the number of circulating EPCs notably lower in LC patients, but the function of circulating EPCs was also remarkably impaired in this patient population.

Previous studies by other authors and those from our group have identified an association between enhanced systemic cytokine levels, inflammatory responses, and cellular apoptosis after myocardial infarction [33,36]. One of the principal findings in the present study is that MCA in circulation was substantially elevated in LC patients than that in healthy subjects. Additionally, late MCA was further increased in Child’s class B and C patients than those in class A. Taking into consideration the fact that chronic liver disease is a process of persistent inflammation, our findings (i.e. MCA) are believed to be the results of chronic inflammatory process in these patients. In this way, these distinctive findings are consistent with those from previous studies [33,36].

In this study, EV bleeding was significantly and independently correlated to an increased number of circulating EPCs. This finding suggests that EV bleeding with acute blood loss may stimulate generation and release of hematopoietic and non-hematopoiectic stem/progenitor cells from bone marrow into the circulation. On the other hand, increased serum level of AST might implicate more severe inflammation and damage in liver parenchyma that might, in turn, suppress the mobilization of EPCs from bone marrow to circulation.

This study has limitations. First, we have no idea regarding the explanation for the finding of chronic hepatitis B as a significant and independent factor associated with an increased number of circulating EPCs in this study. Similar difficulty in explanation is applicable to the negative association between the presence of hepatitis C and a decreased number of circulating EPCs. Second, despite our interesting findings, the sample size of our series is relatively small. All conclusions based on our results, therefore, are tentative and need further support from well-controlled clinical studies of larger scale.

## Conclusion

The results of our study demonstrated that the level, angiogenic ability, and function of circulating EPCs were significantly reduced in cirrhotic patients compared with those in healthy subjects. By contrast, the plasma levels of SDF-1α and circulating MCA were substantially enhanced in LC patients. These findings may highlight the fact that circulating level of EPCs is reduced not only in patients with risk factors of coronary artery disease but also in patients with LC, especially in those patients with decompensated LC.

## Competing interests

The authors declare that they have no competing interest.

## Authors’ contributions

All authors have read and approved the final manuscript. L-TC indicates equal contribution in this study compared with the first author. H-WC indicates equal contribution in this study compared with the corresponding author.
